# Reading Jeffrey C. Alexander, Part II: The theoretical core (and its tensions)

**DOI:** 10.1177/07255136261464663

**Published:** 2026-07-13

**Authors:** Celso M. Villegas, Galen Watts

**Affiliations:** 1 3475 Kenyon College, USA; 2 8430 University of Waterloo, Canada

**Keywords:** Jeffrey C. Alexander, sociological theory, multidimensionality, cultural sociology, postpositivism, civil sphere

## Abstract

In ‘Part II’ of this two-part article, we provide a general schematic with which to introduce the Alexanderian vision of sociology. This vision comprises the following five commitments: (a) a commitment to structural voluntarism and the moral dimension of human action; (b) a commitment to multidimensionality; (c) a commitment to the centrality of meaning in social life, and thus to culture's autonomy; (d) a commitment to a postpositivist conception of social science; and (e) a commitment to democracy as a utopian ideal. In addition to elaborating this vision, we also explore its theoretical tensions and strains, and the way these serve as a fecund source of generative thought and theoretical development − giving rise to new problems, as well as new attempts at resolution.

## Introduction

In ‘Part I’ of this two-part article, we made the case for Alexander's canonization as a classical theorist. However, precisely what the Alexanderian vision of sociology entails − and what it means for the wider discipline − are by no means self-evident. For while ‘Jeffrey C. Alexander’ may have few denotations, it has many connotations. In this, ‘Part II’ of this article, we provide a general schematic with which to introduce the Alexanderian vision to those readers unfamiliar with it, while also helping them to navigate through it. So, having periodized Alexander's life and career in Part I, in Part II we offer our own reconstruction of the *theoretical core* of Alexander's sociology.

It will perhaps be surprising that, despite the terrific depth and richness of Alexander's *oeuvre*, the underlying vision that animates it has remained fairly stable over time (although relative emphases and priorities have certainly fluctuated). This vision, as we see it, comprises the following five commitments: (a) a commitment to structural voluntarism and the moral dimension of human action; (b) a commitment to multidimensionality; (c) a commitment to the centrality of meaning in social life, and thus to culture's autonomy; (d) a commitment to a postpositivist conception of social science; and (e) a commitment to democracy as a utopian ideal.

In what follows, we spend time elaborating the roots and subsequent development of each of these five constitutive commitments. We also explore the tensions, problems and even antinomies, that attend them. Alexander has written (perhaps with regret), ‘Great theories are, in almost every case, fundamentally ambiguous’ (Alexander, 1979a: 345) and ‘social theories are, in fact, rife with confusions and self-contradiction’ (Alexander, 1982d: 272). Knowing him, we doubt he would exempt himself from such statements, and so we think it obligatory to consider the possibility that they might also apply to his work. Of course, one of the unique aspects of Alexander's theoretical development has been his extraordinary sensitivity to the analytic tensions inherent to his own projects − and with this, a dogged persistence to overcome them through a reflexive incorporation and integration of seemingly hostile traditions. Yet if we accept a young Alexander's claim that all theories contain within them certain tensions which can be reduced but never excised (see Alexander, 1979a), then we must assume that Alexandrian sociology is no less implicated.^1^ And so, we spend some time exploring not only the nature of these tensions and strains, but also the ways they serve as a fecund source of generative thought and theoretical development − giving rise to new problems, as well as new attempts at resolution.

## Structural voluntarism and the moral dimension of human action

Since the beginning, the core of Alexander's work has been a principled commitment to voluntarism − that is, to the autonomy or freedom of the human agent. In fact, it is precisely this commitment that roots Alexander to this day in the Parsonian tradition. In his first piece to be published in the *American Sociological Review*, Alexander (1978) elaborates on what he refers to as the ideals of ‘formal’ and ‘substantive’ voluntarism in the work of Parsons. By ‘formal’ voluntarism, he refers to the way that Parsons, following Durkheim (who followed Kant),^2^ conceptualized freedom not as the absence of all constraints, but rather as freedom vis-à-vis material or ‘external’ constraints. For the philosopher Kant (1993 [1785]), human agents can only be said to be free when they act in accordance with rationally-binding normative standards. Durkheim, and then later Parsons,^3^ would revise this Kantian conception of voluntarism sociologically, by treating the individual as comprising distinct social elements (or ‘environments’ (Alexander, 1988a)), and then distinguishing between those which are ‘internal’ to human agents (i.e., subjective, normative ideals) and those which are ‘external’ to them (i.e., rational interests and the objective, material environment).^4^ Crucially, then, for Kant, Durkheim, Parsons and Alexander as well, the existence of human autonomy is fundamentally dependent upon the possibility for nonrational moral action. As Alexander explains in a later essay:

‘There is a profound moralism at the heart of Parsons's theory. His actors are imbued with a desire to be good, and they are understood as trying to conform with principles that express this moral aspiration. Because moral principles are reference points for human action, human beings want their institutions to bear an appropriate relation to them. The fact that human beings make choices is essential here. It is because they have the ability to make choices that normative standards of evaluation become essential’ (Alexander, 1990a: 342).

This commitment to voluntarism, or to a sociological conception of free will, explains Alexander's profound antipathy to sociologies which he interprets as deterministic. For instance, his abiding suspicion of Marxism as a social theory derives from his view that, at the level of theoretical logic, it denies normative or nonrational ideals of any motivating power. That is, Marxist theory presupposes an instrumentally rational conception of action, whereby the calculating behaviour of individuals is ultimately determined by ‘external’ objective conditions (Alexander, 1982b: 203). For his part, Alexander finds this as morally repulsive as he finds it empirically implausible.

Recall that Alexander's eventual rejection of cultural Marxism derived in no small part from his witnessing first-hand an authoritarian turn among his fellow college leftists. Relatedly, he came to the view the far left's embrace of Stalinism, not as an aberration, but as a logical development of the underlying presuppositions of Marxist theory:

‘Super-structural logic cuts both ways, for if ideas are mere reflections of material interests, morals cannot be less so. Morality becomes merely an instrument in the class struggle, divided, in Trotsky's famous phrase, into “their morals and ours.” This radical relativism obviates the individual's moral responsibility for his actions, and the stage is set intellectually for some future conjoining of “humanism and terror,” the logical conclusion drawn by Merleau-Ponty in his famous apology for Stalinism’ (Alexander, 1982b: 203–204).

In turn, it should be no surprise why Alexander took exceptional issue in the 1990s with the growing popularity of Bourdieu's sociology − whom he interprets as an unorthodox successor of the (neo)Marxist tradition (Alexander, 1995). According to Alexander, by ostensibly reducing normative action to strategic interest Bourdieu shares with other Marxist theories a systemic denial of human freedom − and with this, a perennial openness to authoritarianism (see Alexander, 1995: 187).^5^

We can see, then, that Alexander's hostility toward Marxism stems at least as much from what might be called his ‘sociological moralism’ − that is, an ideological commitment to the constitutive role of moral ideals in human action − than from any purely intellectual concerns. That said, as he would surely remind us, ideological commitments comprise only one point along the scientific continuum, and are relatively autonomous from those of theoretical logic (Alexander, 1982a). Alexander's commitment to voluntarism in fact rests equally upon his view that anti-voluntarist theories mire the social theorist in irresolvable performative contradictions. For example, while Marxist theory may reduce all normative ideals to instrumental calculation, the Marxist theorist must exempt themselves from their *own* analysis, or else risk undermining the normative basis of their own social critique. As Alexander puts this conundrum, ‘Marxism is an antivoluntaristic social theory which functions, ideologically, to stimulate active, voluntaristic change’ (Alexander, 1982b: 10).^6^ As a result, Marxist theories always end up appealing to nonrational or normative phenomena, yet are forced to do so in an *ad hoc* ‘residual’ fashion (Alexander, 1982b: 54).

Of course, Alexander's commitment to voluntarism makes him, in principle, equally averse to determinisms of an ‘idealistic’ or ‘cultural’ kind. In fairness, however, Alexander has spent much more time critiquing the shortcomings of ‘materialist’ determinisms. Still, he has at various times voiced criticism of the Durkheimian, Parsonian, structuralist and hermeneutic traditions to the extent that they have reduced action to its normative or cultural elements (see Alexander, 1982b, Alexander, 1982c 1982d, 1987a).^7^ In this way, the romantic expressive spirit of the 1960s has remained a central animating force within the Alexandrian vision from the outset. Owing to his faith in the analytic fact, and concrete possibility, of human freedom, neither a sociology which reduces action to material conditions, nor one that presupposes an ‘oversocialized conception of man’ (Wrong, 1969) is satisfying to him.

At the same time, while a commitment to voluntarism lies at the core of Alexander's vision of sociology, his voluntarism is of a decidedly qualified kind. Indeed, it is his sensitivity to the structural constraints impeding the agency of individuals (perhaps a legacy of his early Marxism), as well as the debts individuals owe to society for their autonomy (deeply Durkheimian in nature (see Durkheim, 2005)), which arguably places him at odds with the antinomian radicalism of the 1960s left, not to mention the purely ‘negative’ ideal of freedom (Berlin, 1969) characteristic of economics and rational choice theory.^8^

Here, again, we can see his debts to Parsons shine through. A central concern of Parsons was to articulate a social theoretical alternative to ‘utilitarianism,’ which he conceived, not as a philosophical tradition, but as a ‘generalized mode of thinking’ (Alexander, 2021a: 155) (typical of capitalist modernity) which assumes an instrumental view of action, and therefore sees a lack of ‘external’ constraints as the sole basis for freedom. For Parsons, utilitarianism, and the theoretical frameworks it sponsors on both the left and right, radically underestimates the social preconditions for human autonomy, as well as the social constraints preventing its concrete realization. And thus, he developed his ‘voluntaristic structuralism’ (Alexander, 1987a: 28) in order to provide a more sociologically-tenable account of individuality and autonomy.

Alexander undeniably carries forward this ‘anti-utilitarian’ spirit of the Parsonian tradition, and in much the same way: by emphasizing − contra those who would reduce human action to rational calculation vis-à-vis objective conditions − the moral and creative dimensions of human action, while simultaneously stressing − in the face of utilitarians who assume a radical subjective freedom − the constraining role of collective order in either its symbolic or material forms (Alexander, 1981, 1984).

Furthermore, it is also this aspect of the Parsonian tradition which places Alexander firmly within the liberal tradition − at odds with either the conservative right or radical left (Alexander, 1979b; Mayhew, 1984). Yet it is crucial to point out that the liberalism that both Parsons and Alexander carry forward is arguably more European (specifically, French and German) than American in provenance (Bellamy, 1992; Seidman, 1983). In America, the liberal tradition has tended toward a more utilitarian, individualist orientation, emphasizing the negative freedom of the individual over and above the constitutive powers of society. By contrast, European liberalism has generally been far more sensitive to the concrete individual's dependence upon institutions and traditions for their sense of self. Arguably it is this grounding in a European-inspired (left-)liberalism that explains some of the difficulties Alexander's work has faced in the American context. By endorsing what might be called a ‘structural voluntarism’ − which ultimately seeks to reconcile a sociological commitment to free will with the existence of collective moral order − Alexander's sociology neither conforms with the implicit individualism of much American sociology,^9^ nor slots neatly into the binary ideological map which defines the American political landscape.

Having said all this, the striking thing about both Parsons’ and Alexander's voluntarism is that they are in key respects bound to the success of the American project especially in its (left-)liberal form (see Alexander, 1987a). And it also explains why, in the post-war era, when Parsons's New Deal liberalism no longer inspired loyalty in the American electorate, the sociological theory it inspired similarly fell from grace. As Alexander observes in his 1978 *American Sociological Review* essay, Parsons’ commitment to voluntarism is not merely *formal* but is also *substantive*, meaning it presupposes the existence of human autonomy not only in theory, but also in historical practice. Indeed, for Parsons the gradual realization of human autonomy is a real-world phenomenon, which he sought to describe and track with his theory of modernization − itself based principally on his studies of the post-war United States. In this way, when Parsons’ writes of ‘institutionalized individualism’ he is not merely offering an empirical description of how structural differentiation leads to increased institutional autonomy, value generalization and the emergence of more autonomous individuals, but is simultaneously articulating an ideological endorsement of the achievements of American society in the post-war era (Alexander, 1978: 186, 1982d: 132; Mayhew, 1984).^10^

It consequently merits considering to what extent the fate of Alexander's vision of sociology is itself bound up with the fate of (left-)liberalism in the twenty-first century − be it in America or beyond.^11^ Like Parsons, Alexander holds the view that human freedom is not merely realizable in theory, but is most likely to be realized in practice in a structurally differentiated liberal society where institutional spheres − the market, the state, the family, the civil sphere, etc. − co-exist in relative equilibrium (Alexander, 2001a). Of course, unlike Parsons, Alexander has not claimed that such an ideal state has been realized, either in the United States or elsewhere (and as we will discuss below, his liberalism is much more chastened than Parsons's, resembling that of Raymond Aron). But it goes without saying that, should sociologists come to doubt the desirability or plausibility of his (left-)liberal ideal, then the Alexandrian vision for sociology may go with it. And although Alexander would of course insist on the relative autonomy of liberal ideology in the reception and assessment of his work, in this moment of mounting frustration with liberalism (see Fukuyama, 2022; Mounk, 2018), perhaps this proposition should be questioned.^12^

Nor is this the only aspect of Alexander's commitments − formal and substantive − to structural voluntarism that might warrant scepticism. While Alexander's sociological revision of the Kantian legacy is, in our view, both laudable and astute, we recognize that not all will be equally persuaded. Alexander's ‘sociological moralism,’ which makes human freedom dependent upon the internalization of normative or nonrational standards, is by no means uncontroversial. For the utilitarian-inclined, this view smacks of traditionalism and paternalism, and risks reifying society while downplaying human agency. Whereas for sociological materialists, this view naively overestimates the role of morality in human action, replacing hard-headed realism with wishful thinking. Furthermore, both groups of critics might draw attention to the way that Alexander has tended over the course of his career to conflate his *analytic* commitment to voluntarism and the moral dimension of human action, with their *concrete* actuality − what we see as reflecting a core theoretical tension in his work.^13^ For while a theorist may be committed to the constitutive powers of nonrational ideals in a formal sense, it does not follow that such ideals are active (or active to the same extent) in every empirical situation (Joas, 1988). Of course, Alexander would undoubtedly agree with this. But he seems at least implicitly unwilling to acknowledge this truth as it relates to the work of those with whom he disagrees. For example, in his scathing critiques of (neo)Marxist sociology, Alexander has a habit of inferring, on the basis of empirical studies, a presuppositional anti-voluntarism (see Alexander, 1982b; Alexander, 1995). Yet to identify instrumental action in any given empirical situation is not, necessarily, to presuppose that all action is thereby instrumentally rational. Again, we are certain that Alexander would agree with this point.^14^ But whether his own work always exhibits such assent remains unclear.

## Multidimensionality

The second commitment that comprises the theoretical core of Alexander's sociology is to multidimensionality. In its initial form introduced in *Theoretical Logic*, Alexander employed this as an evaluative standard, appropriate to his postpositivist conception of social science, to assess the most general presuppositions of a sociological theory − that is, those pertaining to the rationality of action and the nature of social order (see Alexander, 1982a). Animated by the Parsonian quest to produce a total theory, which could unify the ‘warring schools’ of sociology, at this time in his career Alexander believed the greatest impediment to this goal was the rise of narrowly ‘one-dimensional’ social theories − those that emphasize rational action over nonrational action (viz., sociological materialism), those that emphasize nonrational action over rational action (viz., sociological idealism) and those that fail to account for the existence of order (viz., individualist theories). In turn, he developed the regulative ideal of multidimensionality, both as a means to signal what he sought after, as well as to serve as a kind of master key with which to unlock and pick apart the problems with rival theories.

And pick apart he did. Throughout his early phase, Alexander employed this critical standard to take to task not only Durkheim (idealist), Marx (materialist), Weber (inconsistently idealist and materialist) and the later Parsons (idealist), but basically every other sociological theory that was not his own (e.g., Alexander, 1987a, 1988a, 1992). Consider, for instance, that with ‘multidimensionality’ in hand, Alexander could, and did, charge his myriad rivals and critics with everything from failing to acknowledge the existence of conflict (Parsons), failing to appreciate the fact of equilibrium (conflict theorists), failing to give normative interpretation its due (Marx), failing to acknowledge the role of strategic calculation (Durkheim), failing to sufficiently value the meaningful (pragmatists), failing to recognize the instrumental (Geertz), failing to properly value modern rationalization and differentiation (Weber, Foucault), failing to properly criticize it (Parsons, Durkheim) and failing to theorize creative contingency (hermeneutics), while failing to theorize structural constraints (ethnomethodologists) (and this list is by no means exhaustive). In this way, multidimensionality functioned in this period as an extremely effective weapon with which to engage in theoretical combat. Armed with it, Alexander could seemingly rise above the fray and take swipes at those to his left and right, all the while claiming to occupy, if not in reality, then at least in ambition, a kind of archimedean perspective and position.

Of course, putting it this way could be seen to imply that Alexander's espousal of multidimensionality was purely strategic. This is not our view. When Alexander writes at the end of *Twenty Lectures*, ‘Multidimensionality, I believe, is the only position which can explain the social world in a thorough, consistent, and satisfying way’ (Alexander, 1987a: 280), we have no reason to doubt the sincerity of this statement. In fact, it seems apparent that Alexander earnestly believed during this period that a truly multidimensional theory − one which was synthetic vis-à-vis action and which was collectivist vis-à-vis order − could reunify a deeply fragmented sociological community. That is, in the 1980s Alexander saw himself as engaged in an existential-level task of rescuing sociology by means of supplying it with the grand theory it needed to survive.

And yet, given the sincerity of his early-period belief that a multidimensional theory ‘must remain’ sociology's ‘most sought-after goal’ (Alexander, 1982d: 288) it is striking that, as he transitioned into his middle-period, the meaning of multidimensionality seemed to broaden and shift. As Alexander increasingly focused on developing cultural sociology, instead of reconstructing the Parsonian tradition, multidimensionality morphed from being an ideal narrowly concerned with presuppositions about action and order, to encompassing any and all attempts at theoretical synthesis.^15^ And so, we see Alexander in this period striving to integrate not only sociological idealism and materialism, or individualism and collectivism (attempts at synthesis consonant with the original concept), but also subjectivity and objectivity, micro and macro, reason and emotion and pretty well every other sociological dualism.

Here again Parsons's influence is not difficult to detect, but equally important is the example provided by Alexander's supervisor, Smelser. For it was Smelser who provided Jeff with a model of how to integrate seemingly opposing theoretical frameworks in the service of producing a more synoptic perspective.^16^ Arguably, it is this Smelserian penchant for theoretical ecumenism and grand synthesis which remains one of the most enduring and notable features of Alexander's sociology − and which the concept of multidimensionality best captures. Basically, all of Alexander's research projects are the outcome of attempts to integrate the partial insights contained in competing theoretical traditions − be they, symbolic interactionism and structural-functionalism (Alexander, 1988a), structuralism and hermeneutics (Alexander, 2003), practice theory and hermeneutics (Alexander, 2004), or pragmatism and performance theories (Alexander et al., 2006). Indeed, what most stands out about Alexander's near half-century of sociological production is his relentless appetite for theoretical integration, for merging the new with the old, all in pursuit of a more generalized theory. Of course, it is precisely this ambition which the term ‘multidimensionality’ was originally meant to capture. Because theoretical discourse is as much a staple of social scientific debate as empirical argument, a key means by which sociologists demonstrate the superiority of their position is by incorporating the insights of their rivals into their theories. For Alexander, then, it is by seeking a whole that is greater than the sum of its parts that one proves one's mettle.

Yet for all this, it remains an open question whether, in his move to developing the Strong Program in Cultural Sociology, Alexander abandoned his original multidimensional quest. Multidimensionality was initially grounded in the Parsonian desire to produce a general theory that would give equal attention to rational and nonrational forms of action, creative agency and collective order, and the personality, cultural and social systems. By concentrating specifically on the cultural system, and foregrounding the relative autonomy of culture, one could reasonably argue that Alexander is no longer engaged in the task he once felt to be overriding. And if this is true, then it raises the questions: what would the early Alexander think? And perhaps more importantly: what does this tell us about the multidimensional quest in its original form?

Addressing the second question first, there is an intuitive plausibility to the goal of multidimensionality; if social reality is multivalent, then it stands to reason that so too should our theories be. However, this plausibility weakens once the concept is subject to scrutiny. For one, while multidimensionality might be coherent in theory, it is not clear that it can be easily translated into practice. That is, can one really account for *all* dimensions of empirical reality with a single theoretical framework? This seems highly unlikely. Thus, one could argue that the quest to produce a general theory that can subsume social reality, in all its pluralist splendour, is bound to fail. However, assuming this could be achieved, another question emerges: will this in fact produce empirical research that illuminates? It seems a legitimate concern that a theory that seeks to integrate all rival perspectives will soon resemble a stew containing all possible flavours; what it gains in ingredients, it lacks in flavour. Perhaps what we should want, then, are multiple competing theories, each of which brings to light different aspects of the social world? In some respects, this seems to be the view implicit in the Strong Program, for rather than deny its narrow analytic focus on culture, it considers it as a constitutive feature of the programme (see Alexander and Smith, 2010). To be sure, the drawback of this more pluralist approach is that it does nothing to unify the fragmented (or if you prefer ‘multicentric’ (Lizardo, 2014)) character of the discipline. But perhaps this is preferable to instituting a reigning orthodoxy, characteristic of the Parsonian era.

Returning to the first question, it seems to us that from the perspective of the early Alexander, the later Alexander would be seen to have abandoned multidimensionality as it was originally conceived − thereby contributing to, rather than mitigating, the discipline's fragmentation. This is, it must be said, one of the great ironies of Alexander's career; what began as an ecumenical project in theoretical unification evolved into a doctrinaire project that added yet another school engaged in ‘war.’^17^ Furthermore, it seems likely that were the early Alexander to be active on the scene, he would undoubtedly bring the full weight of the critical standard of multidimensionality brought down upon his later self. That is to say, we would likely find the later Alexander taken to task for failing to give sufficient attention, especially in his empirical work, to all dimensions of social reality (though especially those of a ‘material’ or ‘external’ nature).

Nevertheless, in our view this fact should raise as much suspicion of the earlier Alexander as the later one. For in surveying the all-purpose way the early Alexander employed the concept of multidimensionality, it becomes clear that it contained an ambiguity and elusiveness which was rarely noted. Of course, the fact that multidimensionality could be used in these diverse ways is not a problem in itself, but it seems fair to say that, at least in Alexander's hands, the elusive ideal of a *truly multidimensional theory* seems to resemble pretty well anything that he does not approve of. Furthermore, it also bears noting that while multidimensionality is meant to serve as a *theoretical* standard, Alexander has very often wielded it as a means of *ideological* and/or *empirical* critique.^18^ We provided an example of this above, when we discussed the way Alexander imputed to neo-Marxist sociologists a specific presuppositional stance on the basis of their empirical claims. Yet this is a well-worn Alexandrian tactic. For instance, Alexander's core charge against ‘weak’ programmes in the sociology of culture is that they fail to account for culture's autonomy, insofar as they conceive of culture (to use the folk idiom of positivism) as a ‘dependent variable’ (Alexander, 2003). Yet surely in some social circumstances, culture is more a ‘dependent’ than an ‘independent’ variable, and it seems unfair to label a programme ‘weak’ simply because they acknowledge this truism. Similarly, Alexander's (2006) critique of competing conceptions of civil society in *The Civil Sphere* is premised upon the conviction that the civil sphere has relative autonomy vis-à-vis noncivil spheres. But here again, it is not clear whether the disagreement between Alexander and his rivals exists at the level of *theoretical presuppositions* (is the civil sphere autonomous in principle?) or at the level of *empirical reality* (is a given actual-existing civil sphere autonomous?). And so, in his critiques, Alexander has tended to opt for whichever element comprising the standard of multidimensionality permits attack. This habitual vacillation reveals both the genius of this critical standard, as well as its great weakness.

## The centrality of meaning; culture's autonomy

If ever a sharp break occurred across Alexander's sociological career, it was his break away from the Parsonian tradition (in the form of neofunctionalism) in order to develop the Strong Program in Cultural Sociology. Certainly, there are reasons to see this as such; Alexander subjectively experienced this as a crisis, moreover, it did in fact require breaking from Parsons's ‘culturally shallow’ equation of the cultural system with values, in lieu of a more ‘culturally deep’ approach (Alexander, 1987a: 303, 1998). Nevertheless, a longer view of Alexander's intellectual development suggests that the real break took place at Berkeley when he chose to style himself, in the model of Parsons, a systems-theorist. For recall that, as young boy, Alexander was enamoured of literature and its structural properties, and as a Harvard undergraduate in the 1960s, he found himself enthralled, not so much by the empirical precision of cultural Marxism, but rather by its existential and ethical grandeur. It would seem, then, that since his youth Alexander has possessed a profound sense of the importance of *meaning* in human life, or what we might call a ‘cultural musicality.’^19^ This suggests that, rather than his later embrace of the ‘cultural turn,’ it was in fact his earlier foray into the abstract and abstruse structural-functionalism of Parsons which marked a departure − and that, if anything, Alexander's cultural sociology reflects a genuine continuity with his earliest concerns and commitments.^20^

Furthermore, while the theoretical resources had yet to be developed, one can see the seeds of the Strong Program as far back as *Theoretical Logic*. For what is sociological theory, if not a form of culture? And what is theoretical logic if not a kind of cultural structure?^21^ Indeed, there is a sense in which the central thesis of his four-volume tome − that theoretical logic plays an independent role in sociological debate − presupposes something like culture's autonomy. What is more, in the first volume Alexander (1982a) lays out his conception of postpositivism which treats sociological theories as extra-scientific meaning systems with profound implications for social, political and personal life.

Alexander's conviction that meaning is an ineradicable component of social life, and therefore that culture is relatively autonomous of social structure, flows naturally out of his commitments to voluntarism and the moral dimension of human action. Recall that, according to Alexander, human agents are only autonomous or free to the extent that they conform to nonrational or normative (i.e., meaning-full) standards, which must themselves by supplied by culture (Alexander, 1988a). In other words, culture's autonomy is a necessary prerequisite for individual autonomy, just as meaning is a necessary precondition for morality. It is because we are cultural or meaning-making beings that we have the power to change both ourselves and the world.^22^ Additionally, it is because culture is an intersubjective phenomenon that social solidarity is possible, for it is shared codes, symbols, narratives and rituals that provide the bases for community and collective identity. To deny culture's relative autonomy, then, is to deny the possibility of genuine solidarity.

This helps us to see how Alexander's commitments to voluntarism, the morality of human action, the centrality of meaning and the possibility for solidarity lie at the foundation of his reconstructions of the Weberian and Durkheimian traditions − and with them, the Strong Program. Alexander (1989) rejects Weber's pessimistic account of modern rationalization on the grounds that it denies both human agency and the persistence of moral meaning in modernity, and he rejects the early Durkheim's (1984) sociologically materialist account of differentiation on the grounds that it fails to grant relative autonomy to culture. Moreover, his ‘discovery’ and promotion of a ‘late’ Durkheim, whose ‘religious sociology’ treats the sacred/profane binary as constitutive of modern social life (Alexander, 1988b, 2021b; Alexander and Smith, 2003; cf. Weiss, 2019) undoubtedly grows out of these selfsame commitments. At the risk of oversimplifying, we might say that Alexander's polemic against ‘weak’ programmes in the sociology of culture derives almost entirely from his view that these fail to appreciate, not just the constitutive role of meaning in human life, but with this, the significance of culture for human freedom and solidarity.

Ever since his days as a 1960s’ radical, Alexander has harboured the view that (inter)subjective meaning − which is to say, theory, ideas and ideals − has a vital role to play, not only in good sociology, but also in the good life. Thus, ever since the 1980s Alexander's recurring refrain has been that his fellow sociologists are numb to meaning. Indeed, one comes across this charge so often that it can begin to seem formulaic − as if the very existence of the Strong Program in cultural sociology in fact depends upon the boogeyman of ‘weak’ programmes. Intriguingly, Alexander's structural hermeneutics arguably supports this view, given that it conceives of sacred/profane binaries as constitutive of all meaning systems. Thus, we might say that if the Alexandrian vision of sociology itself constitutes a cultural structure, then we can perhaps delineate its underlying codes in the following way:

**Table:** 

**The cultural codes of the Alexander school**	
**Sacred**	**Profane**
Voluntarism	Anti-voluntarism
Moral nonrational action	Strategic rational calculation
Solidarity	Cynicism
Meaning	Materialism
Cultural autonomy	Cultural heteronomy
Strong Program	Weak programmes
Thick interpretation	Thin interpretation
Later Durkheim	Later Marx
Postpositivism	Positivism
Liberalism	Radicalism (left or right)
Democracy	Authoritarianism

Conceiving of Alexander's sociology in this way permits us to see its appeal to those like us. Indeed, we have little doubt that our attraction to Alexander and his vision of sociology derive in no small part from sharing its sacred commitments. At the same time, putting things in these terms also illuminates potential blindspots and omissions.

As we have already noted, there are reasons to think that despite his ecumenical ambitions, Alexander has never treated all traditions equally. His early personal disavowal of Marxism has evidently animated an intense, if not always explicit, hostility to any and all social theories he perceives as mired in the ‘Marxist dilemma’ (Alexander, 1985). For Alexander the left-liberal, Marxism (especially the later scientistic Marx) occupies a polluted or profane position, to be avoided at all costs. Needless to say, this is an important reason why the Strong Program has tended to be charged with sociological idealism by Marxist critics (e.g., McClennan, 2005). Of course, Alexander would argue that this censure flows out of the misunderstandings inherent to Marxist theory (see Alexander, 2005). Though perhaps true, we think a balanced survey of his work suggests this critique is not without some legitimacy.

In their programmatic manifesto for the Strong Program, Alexander and Smith (2003) asserted that, rather than deny the causal role of non-cultural factors in *concrete* reality, they were instead *analytically* foregrounding culture's autonomy (see also Kane, 1991). However, over the years, this strategy evolved into the claim that culture comprises a ‘dimension that is omnipresent in every traditional object of social science’ (Alexander and Smith, 2011: 5), which has since been used to inspire a host of empirical studies that can give the impression that what is truly determining in the ‘last instance’ is culture.^23^

One might see this evolution − from insisting on culture's *analytic* autonomy to insisting on culture's *concrete* autonomy − as evidence of a lack of theoretical consistency. But another way to think about it is as an instance of what Alexander warned about some years ago: ‘To put Weber's famous aphorism into a different context, theoretical logic is not a streetcar that one can get on or off at will. Once riders pay their money, they must stay on the track for the whole trip’ (Alexander, 1992: 214). While the early Alexander may have striven for a multidimensional theory that transcends the dichotomy of idealism/materialism, in his middle-years and later years, he ultimately allied himself with the Durkheimian and Parsonian traditions − both of which he had claimed are carriers of sociological idealism (see Alexander, 1982b, 1982d). It should therefore not surprise us if he inherited the same propensities.^24^

This has been the critique offered, not only by Marxist thinkers, but also by those who are broadly sympathetic with the Strong Program (Hall et al., 2010; Joas, 2005; Kurasawa, 2004). What these critics suggest is that Alexander's vehement distaste for anything that smacks of Marxism (or any other sociological materialism) has ultimately pushed him to endorse, in practice if not in theory, an opposite extreme − that is, insisting not just only on culture's autonomy, but on its supremacy. As the old adage goes, if all you have is a (cultural) hammer, everything looks like a (cultural) nail.

For all this, the question remains whether the charges of ‘idealism’ or ‘materialism’ are a bit exhausted, presupposing an ideal of transcendence whose tenability we should question. Certainly in his early days, Alexander was convinced that he could fashion a theoretical system that would transcend this dualism, not unlike his third phase peers. Should we perhaps chalk this up to the flights of fancy typical of youth? Perhaps the truth is that sociological theories, like meaning systems more generally, are binary and thus are fated to emphasize one set of elements over another? Of course, this is not to say that the goal of synthesis is not worthwhile, surely it is. But perhaps the existence of some ‘residual’ categories is inevitable? Alexandrian sociology, by insisting on the constitutive role of cultural meaning, is always going to foreground cultural factors, just as Marxist sociology, by insisting on the constitutive role of material resources and conditions, is always going to foreground economic factors. To expect that a single theory can, and should, uniformly incorporate all of the insights − normative, theoretical and empirical − of their rivals, is to expect the impossible.^25^

## Postpositivism

The fourth constitutive commitment of Alexander's sociology revolves around his distinctive conception of the nature of social science − which he refers to as ‘postpositivism.’ Alexander has a tendency to define his position in relation to opposing extremes, and here things are no different. In the first volume of *Theoretical Logic*, Alexander (1982a) opens with a scathing critique of what he calls the ‘positivist persuasion’ − which he sees as a dominant force in American sociology. Sociologists guided by the positivist persuasion seek to emulate the natural sciences, and thus emphasize quantification, replication, statistical reduction and the search for laws, and thus deny the role of subjectivity or self-consciousness in the production of sociological knowledge. This persuasion, Alexander maintains, rests upon a misunderstanding of the nature of science. Inspired by historians and philosophers of science such as Kuhn, Polanyi, Lakatos and Toulmin, he introduces the notion of a ‘scientific continuum,’ comprising two distinct ‘environments’ − an empirical and a metaphysical one. Contra positivists, who argue that scientific debate solely concerns the former, Alexander argues that every single scientific statement involves references to *both* environments. That is, ‘all scientific development is a two-tiered process, propelled as much theoretical [metaphysical] as by empirical argument’ (Alexander, 1982a: 30).

While this truth applies equally to the natural sciences, the elements comprising the ‘metaphysical environment’ − that is, presuppositions, definitions, classifications, ideologies and models − play a much more central role in social scientific debate. Indeed, this is why the social sciences are characterized by much more dissensus than the natural sciences. It is also why theoretical discourse and argument play vital roles in the day-to-day production of social science: ‘Empirical underdetermination and theoretical overdetermination go hand in hand. From the most specific factual statements up to the most abstract generalizations, social science is essentially contestable. Every conclusion is open to argumentation by reference to supraempirical considerations’ (Alexander, 1989: 25).

It follows from Alexander's postpositivist conception of social science that there is an ineradicable subjective dimension in all social science. This is, first, because all social science relies on metaphysical or nonempirical commitments, which cannot be validated with reference to the empirical world, but must instead be debated with reference to aesthetic, ethical, political and even existential criteria. The second reason stems from the fact of meaning: ‘It is because meaning is subjective that the method of discovering meaning must be subjective in turn’ (Alexander, 1987a: 301). A positivist sociologist, armed with the most precise of quantifying instruments, will be lost if he or she lacks interpretive acumen. Sensing and sussing out the symbols, codes and narratives that structure the lifeworlds of ordinary human beings requires, among other things, cognitive empathy, a heightened sensitivity to the taken-for-granted and a humanistic disposition. Indeed, this is why Alexander has repeatedly analogized the work of cultural sociology to that of psychoanalysis (Alexander, 2003). There is also a sense in which it shares affinities with literature, art and philosophy, insofar as doing cultural sociology involves combining astute observation of the social world with creative and imaginative reconstruction (see Alexander, 2019a). To recall a point made earlier, if social life can be conceived as a text (Ricoeur, 1971), then doing cultural sociology well requires being both a good *reader* and *writer* of social reality − skills that cannot be separated from the fact of self-consciousness. Hence, Alexander sees himself as carrying out Dilthey's *Geisteswissenschaften*, literally translated as the ‘sciences of the spirit.’

And yet, despite his insistence that social science is inherently subjective, what is most striking about Alexander's postpositivism is that, while it is premised upon a rejection of positivism, naturalism and scientism, it also distances itself from postmodernism's ‘antitheoretical relativism’ (Alexander, 1995: 90) which rejects ideals of rationality, objectivity and explanation. In other words, Alexander sees himself as championing a neo-modernism, which strives to carry forward the Enlightenment dream of reason, albeit in a more tempered cultural sociological mode.

The basis for this endeavour lies in his conception of the nature of reason and its relationship to *theory*. Alexander argues that to aspire to reason is to, in effect, aspire to self-transcendence, to occupy a perspective that is more encompassing that one's own. What Enlightenment thinkers got wrong, then, was supposing that they could achieve a ‘view from nowhere’ (Nagel, 1986), instead of seeking, more realistically, a view from ‘somewhere else’ (Alexander, 1991a: 147). It is this view from somewhere else − ‘a place that is neither the theorist's own personal world nor the world entirely outside it’ (Alexander, 1991a: 147) − which the claim to reason entails, and which, Alexander thinks, social theory makes possible. For what is theory if not an attempt at generalization, which abstracts from the particular in pursuit of the (more) universal? This is why Alexander argues that it is ‘the existence of “theories,” multilayered impersonal worlds that create the conditions of agreement’ (Alexander, 1995: 115) in the social sciences. While sociology may lack a shared Kuhnian paradigm, relative consensus does exist at the level of theoretical traditions and schools. And it is this agreement which makes possible the creation of research programmes (Lakatos, 1968–1969), and with this, ‘normal science’ knowledge cumulation.

Of course, while traditions and research programmes might flourish, it does no good if they cannot speak to, or learn, from one another. It may well be that relative theoretical consensus exists at the level of schools, securing standards of intra-traditional objectivity, but if theoretical schools conceive one another as incommensurable, the possibility of inter-tradition dialogue breaks down, and we have entered the postmodern world of epistemic silos. This is why Alexander emphasizes the centrality of the classics (Alexander, 1987b). It is because disciplines are defined by a shared classical canon that researchers working in different traditions can engage in theoretical discourse. Classics, that is, function as shared reference points for theoretical argument, ensuring a sufficient degree of common knowledge and theoretical consensus to allow for intelligent and productive debate across traditions (Alexander and Colomy, 1998).

Still, the mere existence of classics does not in itself ensure that sociologists belonging to divergent traditions will engage one another in good faith. It is precisely this concern that animates Alexander's commitment to the Enlightenment ‘*project* of universalism’ (Alexander, 1995: 5) − which he sees as rooted in the Western tradition, and more specifically, in the historical achievement that is the modern university (Alexander, 1986b). The problem with postmodernism, then, is that it turns its back on rationality, objectivity, explanation and universalism as cultural ideals, in favour of irrationality, relativism, moral evaluation and pluralism, thereby corroding the cultural and institutional bases for theoretical discourse and scientific argument. In Alexander's rendering, rationality is a distinctive cultural discourse, not an empirical fact, but one which must be protected at all costs. So while he may share the postmodern scepticism toward achieving a universal truth (i.e., a ‘view from nowhere’), he nevertheless believes the Enlightenment goal of achieving a more generalized and encompassing ‘view from somewhere’ is a worthy ideal that must not be abandoned.

This, then, is Alexander's postpositivism. It posits that sociological debate consists of both empirical and nonempirical elements, neither of which can be excised from any given scientific statement. As a result, social science resides in the no man's land between the ‘hard’ sciences and the ‘soft’ humanities, fated to have a foot perennially planted in both camps (Alexander, 2019a). At the same time, Alexander insists on the preservation of rationality, objectivity, explanation and universalism as regulative ideals, culturally- and historically-bound. These ideals, he thinks, are implicit in the nature of the social theoretical enterprise, in that theory by its very nature strives to transcend the subjectivity of the theorist and speak in a more encompassing voice. Thus, Alexander maintains that subjectivity cannot be excised from objectivity, but that this alone does not undermine the project of universalism, for explanatory objectivity in social science entails achieving a perspective that is less neutral than generalized and synthetic.

We are convinced that Alexander's postpositivism has much to offer contemporary sociology, sensitive, as it is, to the philosophical problems attending positivism, as well as the limitations inherent to postmodern relativism. Indeed, in a fashion unlike any other school, it strives to reconcile the rationalist and romantic traditions in sociology, by highlighting the nonrational foundations of rationality, and then using the methods of reason to elucidate the nonrational in turn. It is a great shame, in our view, that this component of his sociological vision has not made more of an impact in the discipline.

Having said this, the tensions inherent to this project seem to us to arise most acutely when the doctrine of postpositivism is moved from the abstract terrain of theory to the concrete terrain of empirical application. For while we agree wholeheartedly with Alexander that the presence of subjectivity does not itself undermine objectivity, where the rubber hits the road will depend on the balance of subjectivity and objectivity in any given scientific statement.

With this in mind, it is useful to repeat a point we made earlier − that one of Alexander's great gifts is his ability to produce emotionally-compelling, aesthetically-pleasing and morally-inspiring *readings*. Indeed, Jeff is a masterly storyteller, whose dramatic accounts of the evolution of sociological theory, of social events and even of his own intellectual development, can cast a powerful spell upon the reader, enchanting as much as persuading. This fact becomes all the more relevant when considered in light of his assertion that a significant dimension of social scientific production derives from ‘the imaginative speculation of the scientist, which is based as much on his personal fantasy as on external reality itself’ (Alexander, 1987a: 6). While it would be unfair to treat this decontextualized statement as more revealing of Alexander's views than any other, we do think it speaks to a tension that lies at the heart of the Alexandrian vision for sociology − a tension whose source lies in Alexander's strong commitment to the autonomy of culture.

Given that humans are, to paraphrase Geertz (1973), animals suspended in webs of significance they themselves have spun, what distinguishes social scientific theories from natural scientific theories is that, through their internalization, they can play an active role in creating the reality they ostensibly describe. This ‘double hermeneutic’ raises the moral and political stakes of all social theoretical production. Further, as Alexander puts it, it is because theories are themselves meaning systems that ‘Sociological theories are not simply attempts to explain the world, but also efforts to evaluate it, to come to terms with broader questions of meaning’ (Alexander, 1987a: 16). It is because of the *existential* character of social theories that the social theorist possesses extraordinary power:

‘Powerful intellectuals create symbolic frameworks that re-fuse fragmented meanings, actions, and institutions. They provide a new horizon of meaning for social actors who, having lost the “sense” of social and cultural circumstance, experience emotional anxiety and existential stress. To command dramatic ideational power, intellectuals must code and narrate newly emerging social realities in a manner that offers salvation’ (Alexander, 2016: 344).

If this seems to assign the social theorist in modernity a position that was once occupied by religious prophets, it is because it does.^26^ It is Alexander's prerogative that secularization has undermined the public role of traditional religious authorities and voices, and replaced them with the reflexive myth-making of the artist and the social scientist: ‘Social theory is, with art, the primary source of self-reflection in the modern world. The product of secularization, it responds to the problems raised by secularization in turn’ (Alexander, 1989: 68).^27^ What this means, in effect, is that Alexander's vision of sociology confers upon social theory immense social, political and even existential responsibility, for it serves not only as a primary vehicle to the development of individual and collective self-knowledge, nor simply as a platform for social criticism (as we discuss in the following section), but also as a source of world-making, enchantment and even personal salvation.

The question all of this raises is whether the postpositivist attempt to achieve a tempered form of objective explanation is compatible with a social theory that conceives of itself as endowed with such grand (normative) ambitions. For it seems plausible to assume that the rationalist goal of producing a more universal (if not truly universal) form of knowledge for the sake of knowledge cumulation, may sit in tension with the romantic goal of (re)imbuing people's lives with transcendent meaning. And yet Alexander's postpositivism theoretically lends itself to both of these endeavours. As we will see, this tension becomes most acute in Alexander's work on the civil sphere.

## Democracy as a utopian ideal

Alexander's passion for democracy can be traced as far back as his participation in Students for a Democratic Society as a Harvard undergraduate. Deeply imprinted by the romantic humanism of the 1960s, Alexander has long embedded in his sociology its dreams of participatory democracy and authentic solidarity. And yet, with the development of the Strong Program, Alexander's conception of democracy has become decidedly more cultural. Thus, against the rationalist accounts of democracy one finds in Rawls and Habermas, Alexander (2006, 2010, 2011a) emphasizes the performative and dramaturgical dimensions of democratic life. The struggle for democracy is waged less in terms of rational persuasion than in terms of symbolic performances. Civil actors, such as activists, politicians and intellectuals are actors in a social drama attempting to convince their audiences of the sincerity and authenticity of their presentations (Alexander, 2017).^28^

Nevertheless, despite reformulating his understanding of democracy to cohere with his cultural sociology, Alexander has never given up his commitment to the democratic ideals of equality, liberty and solidarity − nor his view that sociology can and should work toward realizing them. Indeed, this is yet another aspect of his postpositivism, which willingly foregrounds the ideological aspects and normative aspirations of his sociology.^29^

In his early period, when he was developing neofunctionalism, these took the form of defending − albeit in a more balanced way − Parsons's liberal theory of ‘successful modernity’ (Alexander, 1987a). In fact, one reason for Alexander's move away from Marxism in favour of Parsons's differentiation theory was that he came to think the latter could better diagnose both the promises and potential perils inherent to processes of modernization.^30^ Similarly, his subsequent frustrations with post-Parsonian conflict theories, Bourdieu's field theory, Foucault's accounts of power-knowledge and even Habermas's neo-Marxist concerns about colonization, all stemmed from the view that such criticisms focused on the bad while ignoring the good in modernity.

This desire to hold both of these normative perspectives in mind at once − to balance criticism and faith (Alexander, 1991b) − has been a defining feature of Alexander's sociological vision.^31^ In fact, it is literally built into the Strong Program in Cultural Sociology − which sees all sacred values as dependent upon the existence of their profane opposites; there is no good without evil, morality without immorality, love without hatred, or liberation without oppression. These are hard cultural sociological truths. Thus, in his now decades-long development of civil sphere theory, Alexander has demonstrated through a host of empirical case studies that the discourse of civil society, which he views as essential to democratic life, has been historically employed not only to expand civil rights (Alexander, 2006), emancipate women (Alexander, 2001b), secure social protections for the poor (Alexander, 2007) and integrate racial and ethnic minorities, but also to stigmatize, exclude and de-humanize (Alexander, 2013b). Alexander's takeaway from this is that modernity is ‘Janus-faced’ (Alexander, 2013b: 4) − and that any mature social theory must seriously grapple with this doubleness, rather than wish it away.

However, there does seem to exist at least some tension between a commitment to hard-headed realism of this kind *and* utopianism. And while the language of ‘real utopias’ (Wright, 2012) may serve to soften this strain, we doubt it does away with it entirely. For realism and utopianism entail opposing states of mind and moral orientations. Indeed, this is perhaps why despite his ‘multidimensional’ stance to pessimism and optimism in theory,^32^ Alexander is widely perceived by the sociological community to carry forward the heady American optimism of Parsons. What begins as multidimensional in theory, seems to always end up being one-sided in practice.

Of course, one might contend that the reason for this reputation stems from the hegemony of cynicism and despair within the discipline, which makes even the most moderate of optimisms seem Panglossian. We suspect there is truth in this. But another explanation, which also seems plausible, is that, as Alexander moved away from neofunctionalism and instead embraced a more self-consciously ‘critical’ conception of sociology in the form of civil sphere theory,^33^ his ideological-cum-performative commitment to the utopian ideal of democracy seemed to take priority over other commitments − be they empirical or metaphysical.

One way in which this can be seen is in his gradual shift away from theorizing modernity society as a whole, in the form of differentiation theory, to primarily theorizing one particular institutional sphere − the civil sphere (Watts and Horgan, 2025). In his earlier period, Alexander was of course ideologically committed to democracy, but his rootedness in Parsons's structural-functionalism led him to see this as merely one part of a larger societal whole. And it is precisely this attentiveness to the need for relative balance between spheres − the market, the family, the state, education, religion, etc. − that firmly located Alexander within the liberal tradition (see Mayhew, 1990; Sciulli, 1992). However, with the move toward civil sphere theory, and his embrace of a more self-conscious ‘critical’ theory, we find much less emphasis on the need for balance between spheres and much more on the extent to which societies are becoming more or less ‘civil’ (see Alexander, 2019b).^34^ This renewed focus on democracy as an object of study brings into stark relief a potential strain between the moral, political and even existential role Alexander assigns cultural sociology − and especially civil sphere theory − and the mundane task of accurately representing empirical reality. For given that he sees social theory as performative, as implicitly striving to ‘bring something into being’ (Alexander, 2016: 356), Alexander conceives of civil sphere theory as *actively partaking* in the maintenance and propagation of democracy. Indeed, the civil sphere theorist, by charting the symbolic work of civil actors actually contributes to democratic life, for in doing so they are helping to keep alive the public's faith in democracy.^35^ It follows from this that there seems to exist pressure upon civil sphere theorists to portray in their work a portrait of democratic vitality as opposed to democratic decay.^36^

This tension between empirical ambitions and normative aspirations arguably haunts not just Alexander's civil sphere theory, but the Strong Program more generally. As we have noted, at the core of this programme rests the conviction that, despite the ‘de-fused’ social conditions characteristic of modernity, meaning still matters. Moderns may inhabit highly rationalized institutions, we may be much more reflexive than our premodern forebears and we may be highly aware of the contingent character of ourselves and societies, but none of this undermines our need for myth, for ritual, for the sacred, or for salvation (Alexander, 2021b). In fact, by excavating the social unconscious, and bringing these nonrational forces to light, cultural sociology proves this to be true.

And yet, one might wonder whether the rationalist endeavour of using the methods of reason to elucidate and explain the inner workings of the nonrational, in fact, contributes to romantically re-enchanting the world, or rather functions to do the opposite. For while cultural sociology insists that the world is enchanted, it does so by using the tools of disenchantment (thereby serving as another source of rationalization). And to the extent that the Alexandrian sociologist is successful in spreading their message to the public, they might potentially undermine the very faith they take so seriously as a topic of study.

Let us put this another way: cultural sociology enchants the world not by providing a new meta-narrative to invest our faith in, but rather by illuminating the meta-narratives that ordinary people already have faith in. In this way, it teaches us (sociologists) to have faith in the faith of others. This is in some respects a beautiful and profound message. But it is also, ironically, an elitist one,^37^ and potentially even a dangerous one, insofar as its truths become widely accepted. Consider for a moment what it would mean for a majority of citizens in a liberal democratic society to accept civil sphere theory as an accurate, and complete, account of democratic life. Surely, this would fundamentally alter (and potentially undermine) their civic landscape. For democratic life requires not merely a belief in the importance of *performing* democratic ideals, but a sincere commitment to actually living them. And to the extent that this truth is forgotten, democracy is bound to go with it.

This raises a final tension that lies at the heart of Alexander's vision of sociology − a tension that revolves around *who* the social theorist or cultural sociologist is ultimately accountable to. In his earlier period, the answer to this question seemed clear enough: for Alexander, it was the sociological community to which he felt himself answerable (e.g., Alexander and Colomy, 1998). Indeed, all of his work in general theory was done in service of the discipline of sociology. However, as he shifted into his middle-period, and especially his later-period, the answer to this question becomes less clear. Is the civil sphere theorist chiefly accountable to the sociological community or to the civil sphere itself? In other words, is the civil sphere theorist a civic actor? Or do they belong to a distinct sphere − say, the educational sphere − with its own ideals and interests? And if both, then how does the civil sphere theorist balance these competing commitments? These questions may not have clear answers. But they are surely worth asking nonetheless.

## Notes

1. Alexander remarks, ‘The mark of an outstanding student is his sensibility to the unresolved theoretical tensions in his master's legacy’ (Alexander, 1982b: 7).

2. On the Kantian aspects of Durkheim’s sociology (see Callegaro, 2012 12; Cohen, 1975; Fish, 2013).

3. According to Alexander (1988a), Parsons’s innovation on Durkheim was his use of Freud’s theory of socialization to explain in a sophisticated manner how it is that normative ideals are internalized by individuals.

4. Alexander would revise this neo-Kantian conception in his essay, ‘Action and its Environments’ (see Alexander, 1988a).

5. Needless to say, it is for this reason that Alexander opposes the tradition of Thrasymachus, which denies the possibility of genuine moral action (see Alexander, 2006).

6. Alexander offers a similar critique of the exchange theory of George Homans, which denies normative ideals any causal force (see Alexander, 1986a).

7. Although as we will discuss below, there are grounds for suggesting that Alexander has himself been guilty of this.

8. While many of his 1960s’ peers were embracing either neo-Marxism or more individualist social theories (e.g., exchange theory, ethnomethodology, etc.), he found himself falling under the spell of Parsons's differentiation theory. This quality of being slightly ‘out of step’ with his generation is arguably a recurring feature of Alexander's intellectual career (see Alexander, 2018a).

9. It may seem strange to refer to American sociology as individualistic, but as Alexander has shown, the left in American sociology (the dominant force) tends to espouse implicitly a conception of human freedom which juxtaposes the agency of individuals with the constraining forces of society. For Alexander, this unfortunately fails to appreciate Durkheim's and Parsons’ great insight: that individual autonomy is as much dependent upon society as it is threatened by it (see Alexander, 1984).

10. This is not unlike Durkheim's discussion of ‘moral individualism’ in his famous essay ‘Individualism and the Intellectuals’ − which must be read as both an attempt to empirically describe an emergent moral tradition, while at the same time conferring upon it legitimacy (see Lukes, 1969). On Durkheim as both a sociologist and moralist see (Turner, 1993).

11. As we will discuss below, we think this may explain why Alexander's work has arguably found a more receptive audience outside of the United States.

12. Indeed, his own account of Parsons's rise and fall suggests that ideology can play a central role in determining the fate of a social theory: ‘Only by seeing this ideological dimension of Parsons’ ambition can it be understood how closely the fate of his theory has been tied to social changes in the Western world’ (Alexander, 1987a: 112–113).

13. As we discuss in the ‘Multidimensionality’ section, this is a common tactic employed by Alexander − one which, arguably, flows out of his commitment to ‘multidimensionality’ as an evaluative standard.

14. As we note, this is in fact the same criticism he makes of Parsons (see Alexander, 1982d: 204).

15. There is a sense, then, in which ‘multidimensionality’ came to symbolize the third, post-classical, era in post-war social theory, the collective goal of which was to transcend all manner of theoretical antinomies and binaries.

16. In his co-written tribute to Smelser, Alexander writes, ‘By avoiding the defense against ambivalence, Smelser demonstrated a remarkable ability to take the sword from the hands of those who would destroy him’ (Alexander et al., 2004: 6).

17. Vandenberghe and Côté (2025) make a similar point: ‘Cultural sociology is a strange animal. It is both a general sociology that supposedly covers the whole of society by looking at cultural forms that animate social life, and a special sociology that pays specific interest to a particular sector of society where an agonistic of symbols, meanings and narratives is considered to be salient.’

18. Interestingly, Alexander charged Parsons with doing *precisely* the same thing: ‘Rarely does he [Parsons] argue against a competing theory on the grounds of ideological approbation; he contends only that it is true or false in theoretical/ empirical terms’ (Alexander, 1982d: 195).

19. We might also say (although this is more speculative) that, from an early age, Alexander developed an awareness of the constitutive role of culture in shaping the self. Precisely what precipitated this awareness − for instance, we might imagine his early engagement with psychotherapy and psychoanalysis (see Alexander, 2009) − is an intriguing question.

20. That said, as we have noted already, it was Parsons who insisted on the ‘analytical autonomy of the cultural realm’ (Alexander and Colomy, 1998: 60), and so even here the break was not as dramatic as one might think.

21. In a co-written essay, Alexander critiques Charles Camic for reducing the reception of Parsons's work to structural conditions, and thus failing to appreciate the relative autonomy of theoretical logic (see Alexander and Sciortino, 1996).

22. Alexander explains, ‘sociology and more generally social sciences do not pay enough attention to the internal environment of action. There is a humanistic, ethical, and moral implication of doing so: cultural sociology reminds us that we are alive, for a fundamental aspect of being alive is to be interpretative, to have an imagination’ (Cordero et al., 2008: 529).

23. Consider, for instance, Alexander's claim in *The Performance of Politics*, ‘My argument is that the democratic struggle for power is not much determined by demography or even substantive issues and that it's not very rational either. Political struggle is moral and emotional. It's about “meaning,” about symbolically “constructing” candidates so they appear to be on the sunny rather than the shadowy side of the street’ (Alexander, 2010: xii).

24. It is notable that, in his later period, Parsons remarked that ‘under certain circumstances, “I am a cultural determinist rather than a social determinist”’ (cited in Alexander, 1982d: 212).

25. There also exists a concern among some cultural sociologists about the seeming theoretical dogmatism which attends Alexander's structural hermeneutics. While some strive to mediate and reconcile the warring cultural programmes (Spillman, 2020), Alexander − committed, as he is, to the view that research programmes advance through agonistic competition − seems intent on playing up the differences between them. Here again, we find the recurring tension between Alexander's ostensible desire to unite the discipline of sociology and his steadfast pursuit of theoretical pre-eminence.

26. He writes, ‘there is always an eschatology, not merely an epistemology, in theorizing about social change’ (Alexander, 1995: 10).

27. Needless to say, it is for this reason that Alexander finds the increasing predominance of the positivist persuasion in American sociology − and beyond − as absurd as it is astounding. For positivism perniciously blinds sociologists to the moral and existential nature of their work, to their profound ethical and political obligations and to the real stakes of their vocation.

28. It seems a reasonable question whether Alexander's performative conception of democratic politics is a reflection of the particular media environment of the early twenty-first century. Alexander would likely contend that politics has always been symbolic, and that his theory of social performance merely brings to light what was ever thus. But while this might be true, there are also reasons to think that in this age of mass digital media, where ‘reality’ is increasingly mediated by digitally curated videos and images, Alexander's theories capture something of the distinctiveness of the times.

29. In fact, an important reason for Alexander's eventual disenchantment with Parsons was his later positivist turn (see Alexander, 1982d).

30. In the opening chapter to *Differentiation Theory and Social Change*, Alexander writes, ‘Where Weber posited rationalization and depersonalization as the central characteristics of modernity, and neo-Marxist critical theory followed this path, differentiation theory offers an opportunity to describe modernity in a more ambiguous and, I think, realistic way. It would seem that it is the very pluralization of life-worlds, not their colonization, that creates the distinctive opportunities and pathologies of modern life’ (Alexander and Colomy, 1990: 12).

31. This identifies yet another meaning of ‘multidimensionality,’ as it has evolved in Alexander's work. And in doing so, helps us to see another way in which Alexander has been able to wield this concept to his own benefit. For there is a sense in which insisting that modernity is both good and bad, consisting of ‘frontlashes’ and ‘backlashes’ (see Alexander, 2018b) (to give one example), permits the theorist to *always be right*.

32. Of course, one might argue that Alexander espouses a ‘pessimism of the intellect, optimism of the will,’ but we do not think this is quite right. For Alexander's optimism is as intellectual as it is related to the will.

33. Making explicit what previously remained implicit, Alexander describes civil sphere theory as a site where the ‘normative and empirical sciences meet’ (Alexander, 2006: 3).

34. One potential tension that this discloses is that between Alexander's chastened (left)liberalism and his commitment to democracy as a utopian ideal. Now, Alexander (2006) has sought to subsume these two traditions under the banner of the ‘civil sphere,’ which, in his rendering, institutionalizes both democratic discourse, norms and practices, as well as liberal ideals and institutions. But the truth is liberalism and democracy can, and often, do conflict − both in theory and practice (Fukuyama, 1992; Mounk, 2018). For as Tocqueville (1998) famously recognized, the liberal emphasis on individual rights and freedom has run into conflict with the democratic stress on popular sovereignty and equality. Thus, to the extent that democracy is the lone lodestar of Alexander's critical social theory, his sociology's relationship to the liberal tradition remains somewhat ambiguous. At the same time, alongside his work on civil sphere theory Alexander has produced a number of empirical studies which suggest a continued, if chastened, commitment to liberal modernity (see Alexander, 2011b). The most notable of these would be his essay on the importance of psychotherapy as an institution (see chapter 8 in Alexander, 2013a; see also Alexander, 2009).

35. Of course, this is in some respects true of Strong Program cultural sociologists more generally − whose work ostensibly contributes to re-enchanting the world, by bringing to light the constitutive role of meaning and morality in (late) modernity. But it seems doubly true for cultural sociologists studying democracy, owing to the fact that the autonomy of the civil sphere itself depends upon widespread belief, not only in civil codes, discourses and ideals, but also in their ability to successfully bring about civil repair.

36. This raises, once again, the recurring conflation of *analytic* and *concrete* in Alexander's work: it is one thing to insist on the analytic autonomy of the civil sphere, and another thing to insist on its concrete autonomy in any given empirical situation. And needless to say, it is perhaps this pressure to paint a rosy picture which explains why Alexander has developed a reputation for, at times, being too optimistic in his empirical accounts (e.g., Alexander, 2013c).

37. This brings to light another tension: Alexander's vision of sociology, for all its democratic spirit, is remarkably esoteric, containing very high intellectual barriers, and thus quite Aristocratic in practice. We raise this not to take a cheap shot, but rather to note the seeming difficulties facing any rarefied academic research programme which views itself as uncomplicatedly allied with democracy.
